# Supplementary material for: A feasibility open trial of internet-delivered cognitive-behavioural therapy (iCBT) among consumers of a non-governmental mental health organisation with anxiety
Source: PeerJ. 2013 Nov 28;1:e210. doi: 10.7717/peerj.210 (PMC3845869; doi:10.7717/peerj.210)
Supplement: Supplemental Information 2 [file peerj-01-210-s002.docx]

**Chief Investigator’s Name: Associate Professor Nickolai Titov**

**Information and Consent Form**

***An open trial of guided iCBT education to determine the benefits of the Wellbeing Course for consumers of the Mental Health Association with symptoms of anxiety***

**Introduction and purpose of study**

You are invited to participate in a study of internet-delivered education for consumers with symptoms of anxiety. The primary purpose of the study is to explore whether consumers of the Mental Health Association, NSW Inc., will benefit from an online education course, the *Wellbeing Course*, that is designed to help people learn to manage symptoms of anxiety. The secondary purpose is to determine how acceptable this Course is to consumers. The Wellbeing Course in this study includes online lessons about skills that are common to treating anxiety and homework tasks to facilitate your recovery.

The study is being conducted by Associate Professor Nickolai Titov – Director of the eCentreClinic at the Centre for Emotional Health, Department of Psychology, Macquarie University (Ph 9850 9901, email: [nick.titov@mq.edu.au](mailto:nick.titov@mq.edu.au)), Ms Linda Manoukian - a Psychologist at the Mental Health Association, NSW Inc (Ph 9339 6013, email: [Lmanoukian@mentalhealth.asn.au](mailto:Lmanoukian@mentalhealth.asn.au)), Dr Blake Dear and Mr Luke Johnston, who are both Registered Psychologists, and Mr Matthew Terides, a Research Assistant.

We strongly recommend that every applicant to this Course has recently visited their GP or specialist to rule out physical causes to their symptoms prior to applying to take part in this study.

**What will happen during the study?**

In order for you to have received this Information and Consent form you have telephoned the 1300 794 992 Anxiety Support Line at the MHA, and have been informed about this study. If you decide to participate, you will be asked to complete some online questionnaires about symptoms of anxiety and mood. Eligible applicants will be telephoned by Ms Manoukian (Ph 9339 6013, email: [Lmanoukian@mentalhealth.asn.au](mailto:Lmanoukian@mentalhealth.asn.au)), who will interview you and tell you more about the study, and check that you are likely to benefit from this study.

If you are suitable, you will receive login codes to access the Wellbeing Course, which is provided by the eCentreClinic (www.ecentreclinic.org). You will receive weekly contact from Ms Manoukian, via telephone or email, and she will guide you through the Course. Ms Manoukian is a Psychologist, and has been trained in using the Wellbeing Course and will be supervised by Associate Professor Nickolai Titov and Dr Blake Dear.

Each time you log on to do a lesson we will take that as a sign of continued consent to participate. The education Course will be conducted online. As part of the study you will log on to www.ecentreclinic.org at least every week during the Course. Your Course will last up to 8 weeks, during which we will measure changes in symptom level. To do this we will ask you to complete questionnaires about your symptoms and how they affect you, which will take around 10 minutes to complete. We will measure changes two times throughout the Course: 1) before the course, 2) immediately after the Course. We will also measure symptoms two months after the Course in order to inform us about the medium-term benefits.

We will also measure participation in Lessons, homework, activity on the site, and satisfaction. In total, we expect you will have to commit at least 16 hours over the 8 weeks of this Course.

The material in the Wellbeing Course targets symptoms of anxiety and aims to help people learn to master those symptoms. This Course consists of five Lessons. Each Lesson provides educational material and homework tasks.

- Lesson 1: Introduction: Learn about physical, cognitive and behavioural symptoms.
- Lesson 2: Learn about techniques for managing the thinking (cognitive) symptoms.
- Lesson 3: Learn about techniques for managing the physical symptoms.
- Lesson 4: Learn about techniques for managing the behavioural symptoms.
- Lesson 5: Revise the skills you have learnt. Make a plan for staying well in the future.

Each of the five Lessons contains a printable summary and homework assignment designed to reinforce the material in the lessons.

**Risks and Discomforts**

It is expected that adults with symptoms of anxiety will experience reductions in the severity of their symptoms. It is also hoped that the techniques taught in this Course will be applied by participants after the trial is completed, resulting in long term improved management of symptoms. However, because anxiety often develops in teenage hood and early adulthood, it is likely that most of participants will have experienced distressing levels of emotions for several decades. The Course encourages them to learn and practice techniques for managing symptoms of anxiety and, in the short term, this often leads to an increase in levels of symptoms.

Should you become distressed you can contact Ms Manoukian (Ph 9339 6013, email: [lmanoukian@mentalhealth.asn.au](mailto:blake.dear@mq.edu.au)) or Associate Professor Nickolai Titov (Ph 9850 9901, email: [nick.titov@mq.edu.au](mailto:nick.titov@mq.edu.au)), to discuss this. There are no known discomforts or risks associated with participating in the Course. However, we cannot and do not guarantee or promise that you will receive any benefits from this study.

You are able to withdraw from this study at any stage, without any penalty; your participation is voluntary. If, after beginning this study you do not wish to participate you can email: [lmanoukian@mentalhealth.asn.au](mailto:blake.dear@mq.edu.au), telephone (02) 9339 6013 or fax (02) 9339 6066, and we will arrange your withdrawal. Alternately, you can withdraw without an explanation or without contacting us. Should you withdraw we would recommend that you consider formal treatment for your symptoms, preferably from a mental health professional with experience in treating anxiety. Your GP will be able to advise you about this.

**Costs and Compensation**

There are no direct costs to you in participating, except that you have to provide your own access to the internet and a telephone, and no financial compensation is offered for participation in this study.

**Privacy, Confidentiality, and Results**

Any information or personal details gathered in the course of the study are confidential (*except as required by law)*. You will be provided with a username and password, and to preserve your privacy, only Ms Manoukian will know whether or not you are participating in this study. The results of this study will be reported in a de-identified and group-based format in scientific publications and international conferences.

Immediately after the Course and again 2 months later, you will receive contact from Ms Manoukian summarising your individual results. Six months after the Course you will also be emailed a summary of the overall results, and will be invited to request copies of any published articles when they become available.

Please note that we will ask you to provide details of your GP. We undertake not to contact them, unless we are concerned for your safety.

**Your Consent**

Your decision whether or not to participate will not prejudice your future relations with Macquarie University or the Mental Health Association. If you decide to participate, you are free to withdraw your consent and to discontinue participation at any time without prejudice.

If you have any questions, please feel free to ask us. If you have any additional questions later, Ms Manoukian (Ph 02 9339 6013) or Associate Professor Titov (Ph 02 9850 9901) will be happy to answer them.

Please print and keep a copy of this form, but **please click reply to this email if you wish to participate in this study**.

**CONSENT FORM**

**An open trial of guided iCBT education to determine the benefits of the Wellbeing Course for consumers of the Mental Health Association with symptoms of anxiety**

**Please send this email back to** [lmanoukian@mentalhealth.asn.au](mailto:blake.dear@mq.edu.au) **by clicking the “reply” button. When we receive your email we will regard you as having consented to join the study.**

1. I would like to participate in this study

2. I acknowledge that I have read the Participant Information Statement, which explains why I have been selected, the aims of the study and the nature of my participation and the possible risks and benefits.

3. I have been given the opportunity of asking any questions about my participation and about any risks and benefits.

4. I understand that my GP may be contacted if there are concerns about my safety during the Course.

5. I understand that I can withdraw from the study at any time without prejudice to my relationship to Macquarie University or to the Mental Health Association.

6. I understand that quality assurance is undertaken to continually improve these research projects. I understand non-identifiable data from this project may be used in quality assurance studies not described in this form.

7. I agree that research data gathered from the results of the study may be published, provided that I cannot be identified.

8. I understand that if I have any questions relating to my participation in this research, I may contact Ms Manoukian (Ph 02 9339 6013) or Associate Professor Nickolai Titov (Ph 02 9850 9901), who will be happy to answer them.

If you have any complaints or reservations about any ethical aspect of your participation in this research, you may contact the Ethics Review Committee through the Director, Research Ethics (telephone 9850 7854; email [ethics@mq.edu.au](mailto:ethics@mq.edu.au)). Any complaint you make will be treated in confidence and investigated, and you will be informed of the outcome.
