# Supplementary material for: A feasibility open trial of internet-delivered cognitive-behavioural therapy (iCBT) among consumers of a non-governmental mental health organisation with anxiety
Source: PeerJ. 2013 Nov 28;1:e210. doi: 10.7717/peerj.210 (PMC3845869; doi:10.7717/peerj.210)
Supplement: Supplemental Information 3 [file peerj-01-210-s003.docx]

**Protocol Title**

An open trial of guided iCBT education to determine the benefits of the Wellbeing Course for consumers of the Mental Health Association with symptoms of anxiety

**MQ HREC Ref:** 5201200348

**Version Number:** 1.0

**Date of Protocol:** 5/05/2012

**SYNOPSIS**

Protocol title: **An open trial of guided iCBT education to determine the benefits of the Wellbeing Course for consumers of the Mental Health Association with symptoms of anxiety**

Protocol Version: 1.0

LIST OF INVESTIGATORS

Chief Investigator: Associate Professor Nickolai Titov

Address: Department of Psychology, Macquarie University, NSW 2109, Australia.

Telephone no.: 9850 9901

Fax no.: 9850 8062

Co- Investigator: Dr Blake F. Dear

Address: Department of Psychology, Macquarie University, NSW 2109, Australia.

Telephone no.: 9850 9979

Fax no.: 9850 8062

Associate Investigator: Ms Linda Manoukian

Address: Mental Health Association NSW501/80 William Street, East Sydney

Telephone no.: 02 9339 6013

Fax no.: 02 9339 6066

Research Co-ordinator: Mr Luke Johnston

Address: Department of Psychology, Macquarie University, NSW 2109, Australia.

Telephone no: 9850 9464

Fax no.: 9850 8062

Research Assistant: Mr Matthew Terides

Address: Department of Psychology, Macquarie University, NSW 2109, Australia.

Telephone no: *To be confirmed*

Fax no.: 9850 8062

**Summary**

**Protocol title:** **An open trial of guided iCBT education to determine the benefits of the Wellbeing Course for consumers of the Mental Health Association with symptoms of anxiety**

**Protocol version:** 1.0

**Objectives:** **Primary objectives:** To determine the short and medium term efficacy of the online eCentreClinic Wellbeing Course to MHA consumers, when administered by the MHA.

**Secondary objective:** To determine the acceptability of the online eCentreClinic Wellbeing Course, to MHA consumers.

**Study design:** Open trial design with one group.

**Planned sample size:** The design (see Stat considerations, below) requires a minimum sample size of 15, but 20 will be recruited.

**Selection criteria:** Australian adults aged 18-64 with a principal complaint of symptoms of anxiety (not OCD).

**Study procedure:** Consumers contacted the Mental Health Association (MHA), NSW, telephone support line will be informed about the study. Interested consumers will be sent an Information and Consent Form. Those who return the completed Consent Form will be contacted by an MHA Psychologist who will telephone the applicant and administer a screening questionnaire. Eligible applicants will be sent login codes to access an online education course, designed to help people learn to manage symptoms of anxiety. This evidence-based course, the Wellbeing Course, includes five online lessons and homework assignments, and all participants will receive weekly contact from the MHA Psychologist to provide support and guidance.

Participants will complete questionnaires at pre-treatment, post-treatment, and then at 2 month follow-ups. Participants will also complete two brief questionnaires before each of the 5 lessons.

**Stat considerations**: With respect to power, a sample size of 15 is sufficient (power at 80%, and alpha at 0.05) to detect pre-post within group differences in effect size of 0.7, which is considered the minimum based on our previous studies.

Analyses will be conducted using parametric techniques and intention-to-treat and completer models.

**Duration of the Study:** 12 months

TABLE OF CONTENTs PAGE

1. BACKGROUND 5

1.1 Disease Background 5

1.2 Rationale for Performing the Study 5

2. STUDY OBJECTIVES

2.1 Primary Objective 6

2.2 Secondary Objective 7

2.3 Additional Objectives 7

3. STUDY DESIGN

3.1 Design 7

3.2 Study Groups 7

3.3 Number of Participants 7

3.4 Number of Centres 7

3.5 Duration 7

4. PARTICIPANT SELECTION

4.1 Inclusion Criteria 7

4.2 Exclusion Criteria 8

5. STUDY OUTLINE

5.1 Study Flow Chart 9

5.2 Investigation Plan 10

5.3 Study Procedure Risks 11

5.4 Recruitment and Screening 12

5.5 Informed Consent Process 13

5.6 Enrolment Procedure 13

5.7 Randomisation Procedure 13

6. SAFETY

6.1 Adverse Event Reporting 13

6.2 Serious Adverse Event Reporting 13

6.3 Participant Safety 13

6.4 Data Safety and Monitoring Board - membership and responsibilities 13

6.5 Early Study Termination 14

7. BLINDING AND UNBLINDING 14

8. STATISTICAL CONSIDERATIONS 14

8.1 Sample Size Calculation 14

8.2 Analysis Plan 14

9. STORAGE AND ARCHIVING OF STUDY DOCUMENTS 14

10. REFERENCES 15

11. APPENDICES 17

11.1 Questionnaires 18

11.2 Diagnostic Interview 27

1. BACKGROUND

## Disease Background

Anxiety disorders affect approximately 14% of the adult population annually and are associated with disability, reduced quality of life, and chronicity. Anxiety can be treated effectively with pharmacotherapy and psychological treatments such as cognitive behavioural therapy (CBT). However, less than 50% adults with anxiety seek treatment each year. Barriers to accessing treatment include the direct and indirect costs of treatment, the limited number of trained mental health professionals, and stigma. [1].

## Rationale for Performing the Study

Internet-delivery of CBT (iCBT) is one strategy for improving access to evidence-based treatment for anxiety disorders [2-4]. The CI and his team have now completed 30 trials of iCBT with more than 2200 Australian adults with depression and anxiety disorders. All trials have resulted in clinical outcomes at least comparable to those obtained from good face-to-face treatments, with high levels of acceptability to consumers. The Mental Health Association (MHA), NSW, provides education, advocacy, and support to consumers with anxiety. They would like to assist consumers to access evidence-based resources.

The planned project is a collaboration between the MHA and eCentreClinic at the Centre for Emotional Health (CEH), Macquarie University. This initial study will examine the feasibility of the MHA administering the online interventions developed at the eCentreClinic. This will be achieved through this initial trial, which utilises an open-trial design with a small number of participants (n=20), who will be recruited and managed by an MHA Psychologist, using software and an online course developed by the eCentreclinic.

**Outcome Measures:** Primary and secondary outcome measures will be administered.

The primary outcome measure is:

- Generalized Anxiety Disorder-7 Item Scale (GAD-7) [5]: Measures general symptoms of anxiety. The GAD-7 is sensitive to symptoms of anxiety disorders.

The secondary outcome measures are:

- Patient Health Questionnaire-9 Item (PHQ-9) [6]: Measures symptoms of major depressive disorder.
- Kessler 10-Item (K-10) [7]. Measures symptoms of psychological distress.
- Treatment satisfaction measures [developed by the researchers]: Measures satisfaction with treatment.
- The Things You Do Questionnaire [developed by the researchers]: Measures frequency of adaptive behaviours performed by a participant in the previous week.

In addition, the GAD-7 and PHQ-9 will be administered at the beginning of each lesson to monitor anxiety and mood.

# STUDY OBJECTIVES

## Primary Objectives and hypotheses and endpoints

To determine the short and medium term efficacy of the online eCentreClinic Wellbeing Course to MHA consumers, when administered by the MHA.

These objectives will be achieved by comparing symptom levels (primary and seconday outcomes measures) at each timepoint.

The main hypothesis is that

1. Large effect sizes will be found pre-post, with gains sustained at 2-month follow-up

The Primary Endpoint coincides with the post treatment questionnaires at week 9.

The Secondary Endpoint occurs at 2-month follow-up.

## Secondary objectives

To determine the acceptability of the online eCentreClinic Wellbeing Course, to MHA consumers. This objective will be achieved by measuring consumer satisfaction at 9-weeks (1-week post-treatment).

## Additional objectives

To determine the feasibility of the MHA administering online Courses developed by the eCentreClinic.

# STUDY Design

## Design

Open trial design, n=20.

## Study groups

This design comprises only one group.

## Number of participants

20 in total (1 group).

## Number of centres

2, the eCentreClinic, Centre for Emotional Health, Department of Psychology, Macquarie University, and Mental Health Association (MHA), NSW.

## Duration

1 June 2012 - 30 May 2013 inclusive of intervention, 2 month follow up and write up of results. The Primary Endpoint will be the end of active intervention (i.e., 9 weeks after beginning the Course); the Secondary Endpoint will be at the 2 month follow up (i.e., 2 months after completion of the intervention).

# Participant selection

## Inclusion criteria

(1) Resident of Australia,

(2) 18-64 years of age,

(3) not currently participating in CBT for target symptoms,

(4) a score above 4 on the GAD-7 (indicating at least mild anxiety),

(5) provides informed consent,

(6) access to a computer, printer, and internet

## Exclusion criteria

(1) Severe depression (i.e. score > 22 on PHQ-9), suicidal ideation or plan (based on interview),

(2) suicidal intention or plan,

(3) principal problem not anxiety,

(4) principal problem are symptoms of OCD,

(5) acute psychosis.

# Study OUTLINE

## Study Flow Table

| **Dates** | **Phase** | **Activities** |
| --- | --- | --- |
| June-July 2012 | Recruitment | Eligible applicants recruited by MHA |
| August 2012 | Intervention | Participants begin intervention (8 week Course).  Complete outcome questionnaires at pre-intervention  Complete GAD-7 and PHQ-9 before each lesson  Weekly contact from MHA Psychologist |
| October | Post-Intervention | Participants complete intervention.  Complete outcome questionnaires at post-intervention  Telephone feedback from MHA Psychologist |
| December | 2-month follow-up | Complete outcome questionnaires at 2-month follow-up  Telephone feedback from MHA Psychologist |
| February | Write-up | Write up of results and submission of manuscript for publication |
| May | Project Close-Out | Submit final report to MQ HREC  Submit reports to MHA and eCentreClinic |

## Investigation plan

An open trial, with 1 group (n=20).

This design tests the short and medium term efficacy of the online eCentreClinic Wellbeing Course to MHA consumers, when administered by the MHA.

MHA consumers aged 18-64 years who telephone the 1300 794 992 Anxiety Support line, will be informed about the aims of the study, and invited to apply. Those interest will be provided with more information and then sent an informed consent form, and asked to complete and return this. Those who complete the form will be telephoned by a Psychologist at the MHA who will provide more details, and administer the Patient Health Questionnaire-9 Item and Generalised Anxiety Disorder-7 item questionnaires. Applicants with scores too high or too low on these questionnaires will be automatically excluded with clear information about where to get treatment, while eligible applicants will be asked to provide demographic details and contact details for their GP.

Eligible applicants will receive an email confirming their participation and start date of the course. On the start date they will receive login codes and instructions to begin the 8-week course. The first part of the first lesson includes standardized questionnaires of anxiety, depression and disability. Course content includes evidence-based principles of managing anxiety and depression. Each of the five lessons contains a printable homework assignment designed to reinforce the material in the lessons. Participants will receive weekly telephone calls or emails from trained and supervised clinical staff at the MHA who will provide support, encouragement and will answer questions about the content of the Course.

Participants will be encourage to log at least weekly to review course materials. They will be asked to complete the GAD-7 and PHQ-9 before each lesson and all questionnaires at week 9 (i.e., at post-education) and again at 2 month follow up. At post-treatment and follow up, participants will receive a telephone call from the MHA Psychologist summarising their individual results. Post 2-months, they will be emailed a summary of the overall results, will be invited to request copies of any published articles when they become available and will be provided information about how to access further treatment should they want it.

The intervention Course will be delivered via the website [www.ecentreclinic.org](http://www.ecentreclinic.org). This Course consists of 5 online lessons, homework assignments, and weekly support, conducted over an 8 week period:

1. *Lesson One:*
   1. Learn about the physical, cognitive and behavioural symptoms of anxiety and depression Learn about the principles of CBT
2. *Lesson 2:*
   1. Learn about the relationship between the way you think and feel
   2. Begin monitoring and challenging unhelpful thinking patterns.
   3. Examples based on people with symptoms of anxiety and depression.
3. *Lesson 3:*
   1. Learn about physical symptoms and managing these with de-arousal and activation techniques
   2. Assertiveness skills
   3. Examples based on people with symptoms of anxiety and depression.
4. *Lesson 4:*
   1. Learn about behaviours (low activity and avoidance) that make symptoms worse, and how to challenge those behaviours
   2. Examples based on people with symptoms of anxiety and depression.
5. *Lesson 5:*
6. Learn about problem solving, relapse prevention and staying well
7. Examples based on people with symptoms of anxiety and depression.

At the beginning of the Course all participants will complete the outcome measure questionnaires. The questionnaires being administered in this study are all valid and reliable clinical and research measures.

All participants will complete a second entire set of questionnaires at the post-treatment point (Primary Endpoint), and at the 2 month follow-up (Secondary Endpoint).

In addition, brief measures of anxiety and depression (the GAD-7 and PHQ-9) will be administered at the beginning of each lesson to monitor anxiety and mood. We will also encourage feedback from participants at every stage of the project to determine how to improve the content and delivery of Course.

## Study Procedure risks

It is expected that adults with symptoms of anxiety will experience clinically significant reductions in the severity of their symptoms. It is also hoped that the techniques taught in the Courses will be applied by participants after the trial is completed, resulting in long term improved management of symptoms. However, because anxiety often develops in teenage hood and early adulthood, it is likely that most of participants will have experienced distressing levels of emotions for several decades. The Course Course encourages them to learn and practice techniques for managing symptoms and, in the short term, this often leads to an increase in levels of symptoms.

Our priority is to support participants to stay safe. Steps for doing this include:

At Application:

Applicants with severe symptoms of depression or suicidal ideation or plan (total score of >23 on the PHQ-9, a reliable and valid measure of the symptoms of depression, and interview by a Psychologist), will not be eligible to participate, and will be provided with written contact details of how to access urgent support and will be strongly encouraged to do so. Applicants assessed as having a principal disorder not targeted by the Courses will not be eligible for this study, as they are unlikely to benefit from the Course. Instead, they will be encouraged to consider other treatment options including accessing their GP or local services, and informed about more appropriate iCBT Courses which may target their specific problem.

During the telephone interview, all applicants are screened for risk of suicide or self-harm. Those at risk will not be eligible to participate, as we believe they require more immediate and intense support than can be provided via the Internet. Instead, they will be assisted to access urgent support from specialist crisis management services and this support will be followed up with written information.

All applicants will also be informed (verbally and in writing) of steps to take if they feel emotionally distressed during the study, including the contact details of the researchers, the importance of contacting their own GP, and contact details of emergency crisis services. Applicants must also provide contact details for their GP or GP Practice, who the MHA Psychologist will only contact if sufficiently concerned about the participant (this is explained to participants verbally, and in the Participant Information Sheet). We do not contact GPs as a standard protocol, as many participants have said that they prefer their participation to remain confidential.

During the Trial, Post-Treatment, and Follow-Ups:

At pre, post-treatment, and at follow-up (2 month), and before each of the 5 lessons, all participants complete a reliable and valid measure of the symptoms and severity of depression (the Patient Health Questionnaire-9 Item; PHQ-9). An increase of 5 or more points is considered an indicator of deterioration, and if this occurs, it will trigger telephone and email contact from the Psychologist. In the event of having contact with a participant who has deteriorated, a Psychologist will make 3 attempts to co-ordinate a telephone assessment to determine risk and develop a management plan. They will also email the participant with details about accessing crisis services.

All participants will also be provided with written information about contact details for emergency services (this information is also included in the footer of all email correspondence), and written details about staying safe in mental health emergencies are included in several locations in the Course materials.

Members of the research team will meet at least weekly to discuss progress. The Chief Investigator will monitor overall progress weekly and will take overall responsibility for this trial.

## Recruitment and screening

Recruitment

Participants will be recruited from MHA consumers over a 2 month period. MHA consumers aged 18-64 years who telephone the 1300 794 992 Anxiety Support line, will be informed about the aims of the study, and invited to apply. Those interest will be provided with more information and then sent an informed consent form, and asked to complete and return this. Those who complete the form will be telephoned by a Psychologist at the MHA who will provide more details, and administer the Patient Health Questionnaire-9 Item and Generalised Anxiety Disorder-7 item questionnaires. Applicants with scores too high or too low on these questionnaires will be automatically excluded with clear information about where to get treatment, while eligible applicants will be asked to provide demographic details and contact details for their GP.

Participants will be recruited over 2-months, or until 20 people are recruited.

## Informed consent process

Potential applicants are instructed to read the Participant Information Form and then decide whether to complete and return the Consent Form. Additionally, each time a patient logs on to complete a lesson will be taken as a sign of continued consent to participate.

# Safety

## Adverse event reporting

Regular team meetings will be conducted to monitor any difficulties participants may be having and ways of best dealing with these difficulties. Serious adverse events will be reported to the Ethics Committee.

## Serious adverse event reporting

Serious adverse events will be reported to the MQ HREC via SAE documentation and emails. Such information will also be reported to the MHA CEO, Mrs Elizabeth Priestley, and the Centre for Emotional Health Director, Professor Ron Rapee, and discussed at additional meetings of the researchers.

## Participant safety

Our priority is to support participants to stay safe. Steps for doing this include:

At Application:

Applications with severe symptoms of depression and/or suicidal ideation/plan will not be eligible to participate, and will be provided with written contact details of how to access urgent support and will be strongly encouraged to do so. All applicants will be informed of steps to take if they feel emotionally distressed during the trial, including the contact details of the researchers, the importance of contacting their own GP, and contact details of local emergency services.

During the Trial, Post-Treatment, and Follow-Ups:

During the trial participants will complete a weekly measure of anxiety symptoms (GAD-7) and depression (PHQ-9) to monitor increased levels of either of these symptoms. If symptoms significantly deteriorate or do not improve, the individual will be contacted as soon as possible by the MHA Psychologist.

Significant deterioration in mood or elevated distress triggers protocols involving the MHA Psychologist contacting the participant to discuss their emotional state, and to develop a management plan, which is then documented and followed up as per eCentreClinic policy. The MHA Psychologist is a fully registered Psychologist trained to manage consumers at risk.

Risk will be monitored through the weekly administration of reliable outcome measures which will monitor symptoms of depression and suicidality as well as symptoms of anxiety.

## Data safety and monitoring board

## Data safety will comply with the National Statement on Ethical Conduct in Human Research. Only the responsible clinicians, delegated research assistants, and Chief Investigator listed on this application will handle data.

## For the purposes of this study, the eCentreClinic will not collect personal identifying information. This will be collected by the MHA and stored in a password protected file in a password protected network.

## Early termination

If a participant wishes to withdraw from the study once it has started, he or she can do so at any time without having to give a reason. This process will be supervised by the MHA Psychologist. Upon early withdrawal, all participants will be provided with encouragement to access additional services via their GP. Additionally, all participants will be given the opportunity to provide feedback about their current symptoms. If participants withdrawing from the Course do report a deterioration of their symptoms, they will be contacted by a Psychologist or Chief Investigator to discuss access to additional services. If participants do not choose to provide feedback about their symptoms, but the investigators have concerns about the participant's condition, we do have consent from all participants to contact their GP.

# Blinding and unblinding

Study is not blinded.

# STATISTICAL CONSIDERATIONS

## Sample Size Calculation

With respect to power, a sample of 15 consumers, with power at 80% and alpha of 0.05, will be sufficient to detect a pre-post effect size difference of 0.7, which is the minimum difference expected based on previous studies at the eCentreClinic. However, to control for attrition, 20 participants will be recruited.

## Analysis Plan

Analyses will be conducted using intention-to-treat and completer models.

# Storage and archiving of study documents

Throughout the study, research investigators at the eCentreclinic will have access to non-identified data, while the MHA Psychologist will be able to identify participants. During the data analysis phase, non-identifiable data (i.e. coded data) will be used. At study completion, non-identifiable data will be written to a password protected database. To maintain participant privacy the eCentreClinic operates using a Secure Socket Layer to provide secure encryption of the eCentreClinic data. Additionally the database is firewall protected. Staff associated with the project are aware of, and will adhere to, the National Statement on Ethical Conduct in Human Research [8].

For the purposes of this study, the eCentreClinic will not collect personal identifying information. This will be collected by the MHA and stored in a password protected file in a password protected network.

# REFERENCES

1. Slade, T., Johnston, A., Oakley-Browne, M.A., Andrews, G., & Whiteford, H. (2009). National Survey of Mental Health and Wellbeing: methods and key findings. *Australian and New Zealand Journal of Psychiatry, 43,* 594-605.
2. Titov, N. (2011). Internet-delivered psychotherapy for depression in adults. *Current Opinion in Psychiatry, 24,* 18-23.
3. Andersson, G., & Cuijpers, P. (2009). Internet based and other computerized psychological treatments for adult depression: A meta-analysis. *Cognitive Behaviour Therapy, 38*, 196-205.
4. Cuijpers, P., Marks, I. M., van Straten, A., Cavanagh, K., Gega, L., & Andersson, G. (2009) Computer-aided psychotherapy for anxiety disorders: A meta-analytic review. *Cognitive Behaviour Therapy, 38*, 66-82.
5. Löwe B, Decker O, Müller S, Brähler E, Schellberg D, Herzog W, & Herzberg P Y. (2008). Validation and standardization of the Generalized Anxiety Disorder Screener (GAD-7) in the general population. *Medical Care, 46,* 266-274.
6. Kroenke K, Spitzer RL, Williams JBW. (2001). The PHQ-9. Validity of a brief depression severity measure. *Journal of General Internal Medicine, 16*:606–613.
7. Kessler, R. C., Andrews, G., Colpe, L. J., Hiripi, E., Mroczek, D. K., Walters, E. E., & Zaslavsky, A. M. (2002). Short screening scales to monitor population prevalences and trends in non-specific psychological distress. *Psychological Medicine,* *32*, 959-976.
8. National Health and Medical Research Council (NHMRC) (2007). *National* *Statement on Ethical Conduct in Human Research*. Canberra, NHMRC.

# APPENDICES

11.1 Questionnaires

Questionnaires to be included in the project are:

- PHQ-9: Patient Health Questionnaire – Nine item, (Kroenke, Spitzer, & Williams, 2001)
- GAD-7: Generalized Anxiety Disorder-7 Item Scale (Spitzer, Kroenke, Williams, & Löwe, 2006)
- Things You Do Questionnaire (developed by the researchers)
- Recruitment Process and Interview Protocol


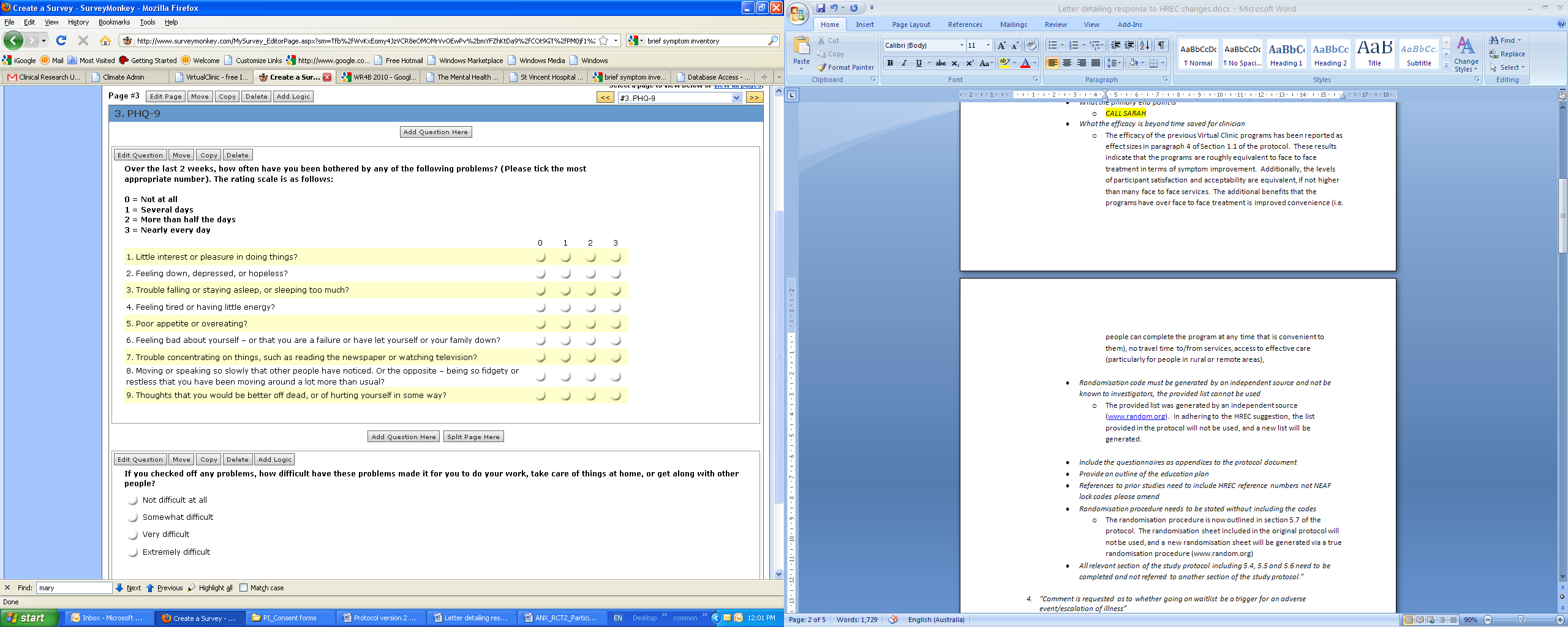
**Patient Health Questionnaire-9 Item (PHQ-9)**


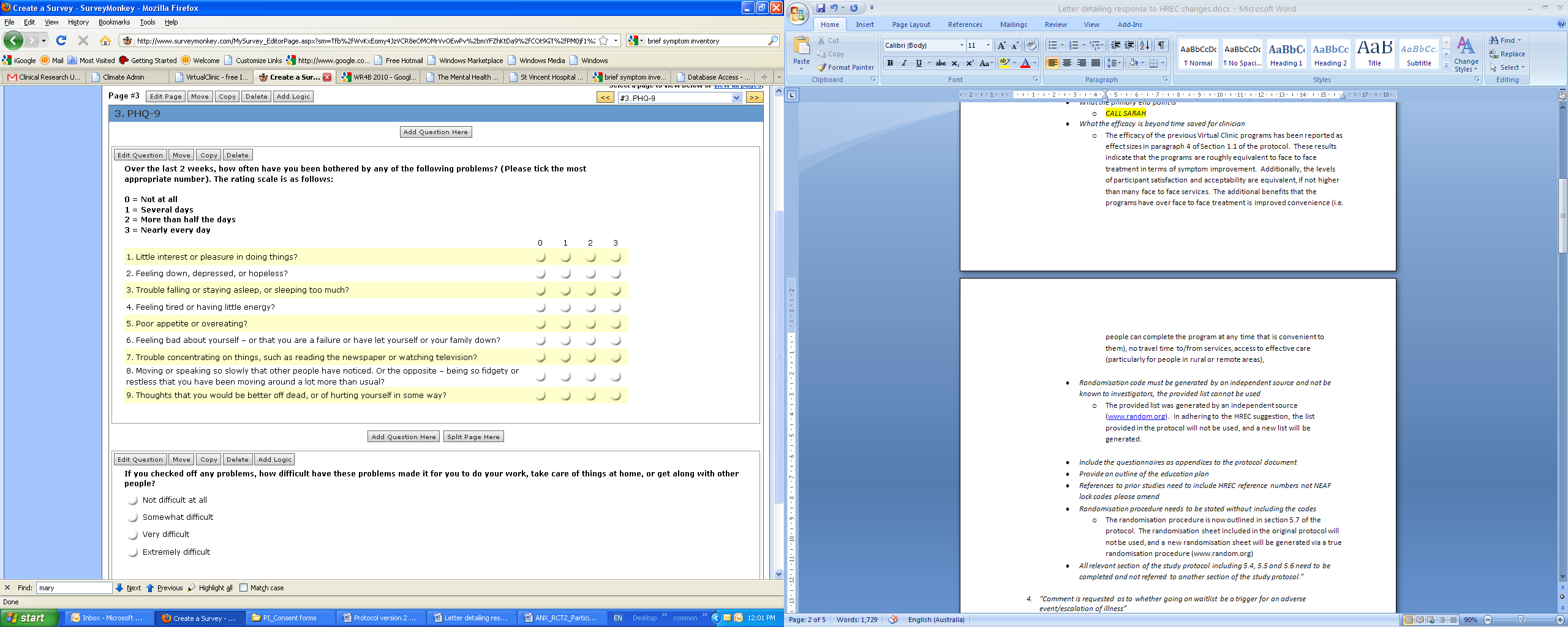
**Generalized Anxiety Disorder-7 Item Scale (GAD-7)**


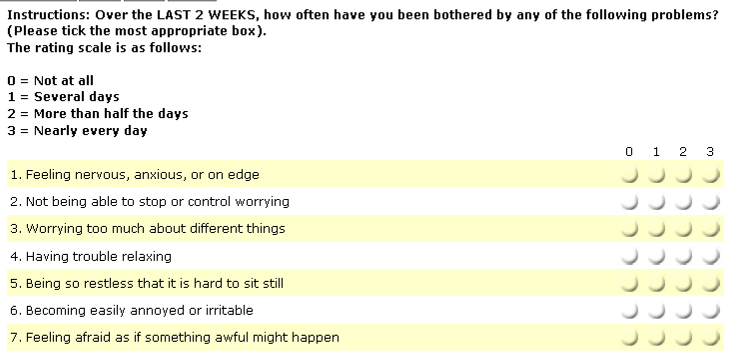


| Things You Do Questionnaire | | |
| --- | --- | --- |
| IN THE PAST WEEK, how many times have you: | | |
| **Response Options**  0 = Never  1 = Once  2 = Twice  3 = Three times  4 = Four or more times | | |
| 1 | Attended a social activity (e.g., meeting a friend)? | 0 1 2 3 4 |
| 2 | Praised yourself when you did something well? | 0 1 2 3 4 |
| 3 | Ate 2 healthy meals in a day? | 0 1 2 3 4 |
| 4 | Did something you enjoy (e.g. a hobby or recreational activity)? | 0 1 2 3 4 |
| 5 | Had something to look forward to each day? | 0 1 2 3 4 |
| 6 | Reminded yourself that you can only do your best? | 0 1 2 3 4 |
| 7 | Did something that you initially wanted to avoid? | 0 1 2 3 4 |
| 8 | Had at least 30 minutes of exercise? | 0 1 2 3 4 |
| 9 | Talked your problems through with a friend or family member? | 0 1 2 3 4 |
| 10 | Talked yourself out of negative thinking? | 0 1 2 3 4 |
| 11 | Had a good night’s sleep? | 0 1 2 3 4 |
| 12 | Did something to help you to relax (e.g., practiced breathing exercises, read a book, surfed the web, etc.)? | 0 1 2 3 4 |
|  |  |  |
|  |  |  |
|  |  |  |
|  |  |  |
|  |  |  |

## Recruitment Process and Telephone Interview

**Summary of Process**

1. Ethics has been approved
2. Awaiting confirmation of Linda's registration as Psychologist
3. Nick to register trial on ANZCTR
4. Once registration confirmed, MHA to: Email potential applicants with Information and Consent Form. Specify timeframe for response before closing of applications.
5. MHA to contact consenting applicants to conduct telephone interview (below).
6. Aim of telephone call:
   1. Check inclusion criteria: Resident of Australia, 18-64 years of age,
   - Not currently participating in CBT for target symptoms,
   - A score above 4 on the GAD-7 (indicating at least mild anxiety),
   - Provides informed consent,
   - Access to a reliable internet connection
   1. Check exclusion criteria:
   - Severe depression (i.e. score > 22 on PHQ-9)
   - Suicidal intention or plan,
   - Principal problem not anxiety,
   - Principal problem are symptoms of OCD,
   - Acute psychosis
   1. Conduct risk assessment and manage safety, as required
   2. Provide information about study
7. Refer Ineligible applicants to be referred to appropriate services, and send email confirming directions
8. Successful applicants to receive email confirming start date of course
9. Aim for 15-20 eligible participants
10. Linda to visit eCC Friday before course begins to create usernames
11. Linda and Blake to login on start date to test dummy accounts.
12. Blake then launches course (start emails sent to participants + Step by Step Guide)
13. Linda to attend supervision sessions (for this Course) at eCC at mutually convenient times (possibly Tue/Thur)
14. Nick/Blake to modify materials to co-brand with MHA: Require permission from MHA for name/signature to use on emails, and MHA logo.
15. Blake to send spreadsheet to Linda
16. Linda to keep spreadsheet at MHA, password protected.
17. Aim to start trial in July, with 2-month follow-up finished November, submit December 2012.

**MHA Open Trial Telephone Interview**

- Hello it’s XXXXX calling from **the MHA …**

1. I’m calling to complete your application for the MHA online anxiety course. To complete your application I need to ask you some questions about your anxiety and your mood. These will probably take 15 minutes to complete, do you have the time right now?
2. First, I want to thank you for applying to our MHA Wellbeing Course. I know it’s quite difficult for most people to apply / takes a lot of courage to apply.
3. I’ll be doing a lot of the talking for this call, but please stop me anytime to ask questions or clarify anything. Is that ok?
4. To start I need to ask you some preliminary questions to check that the course will be suitable for you.

| 1. | Part of the criteria for the course is that you have reliable access to the internet. Do you have reliable access? | Yes | No |
| --- | --- | --- | --- |
| 2. | The internet course is an education course. It is designed to teach people how to manage symptoms of worry, and stress, and panic and anxiety. Does this sound suitable to you? | Yes | No |
| 3. | The internet course is designed for people for whom anxiety is the main concern. Are symptoms of anxiety your primary concern? | Yes | No |
| 4. | The internet course is not designed for people who have symptoms of OCD. Are symptoms of OCD your primary concern? | Yes | No |
| 5. | The internet course is not designed for people with acute psychosis, who are hearing voices, seeing things that other people don't, or believe things that other people don't. Do you have those symptoms? | Yes | No |
| 6. | The Course will begin on XX and will take 8 weeks. Have you got anything planned (e.g. moving house, surgery, holiday) that will interfere with your participation in the Wellbeing Course for more than 2 weeks over that time? | Yes | No |

**IF NOT SUITABLE:**

**Reason:** Unfortunately, this Course will not be suitable for you. *Refer to other sources.* ***Encourage:*** *I really encourage you to go to your GP for a referral to a psychiatrist or clinical psychologist – Medicare does cover 12 sessions per year with these health professionals. OR, other.*

**IF SUITABLE:**

“Now I’m going to start by asking you some questions about mood” (Commence PHQ-9)

**Patient Health Questionnaire-9 Item (PHQ-9)**


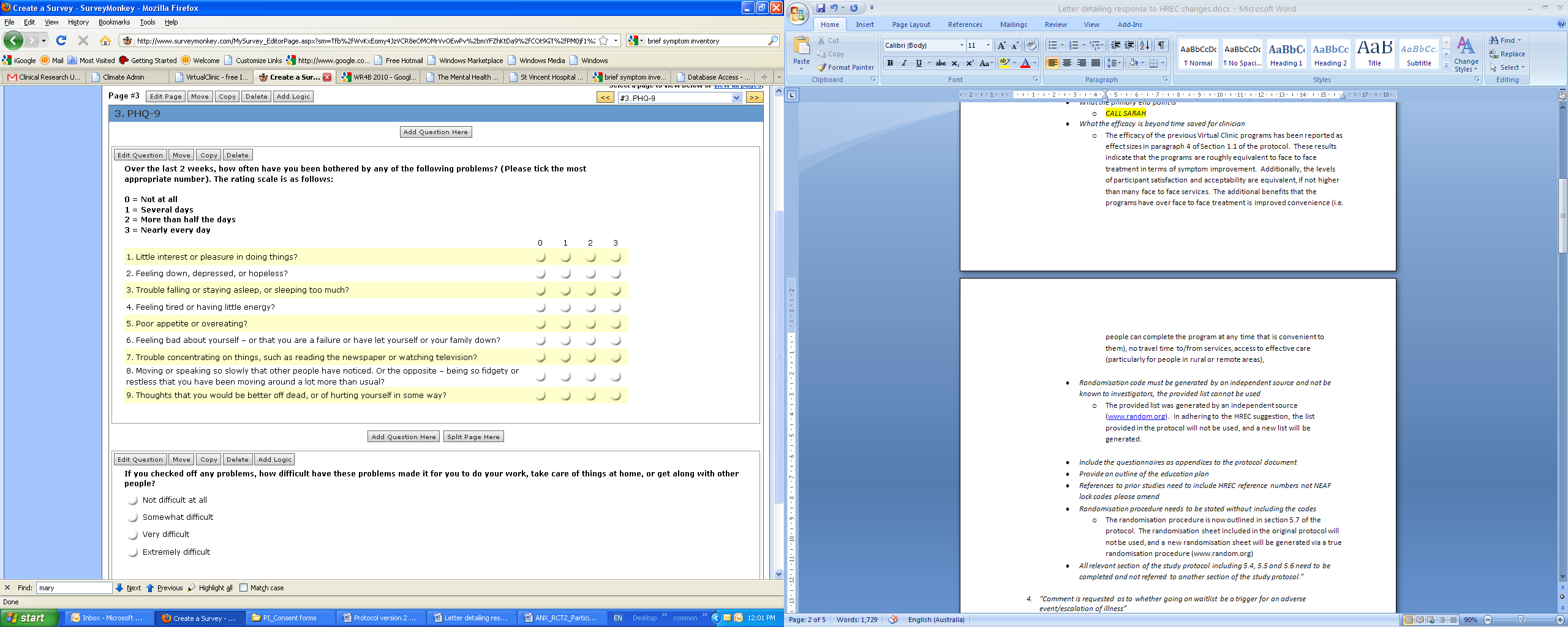


**Exclusion: If Total score > 22, immediately and conduct safety assessment (next page) and exclude. Else, administer GAD-7**

**IF NOT SUITABLE:**

**Reason:** Unfortunately, your symptoms of low mood or depression are really high, and we don't think you will benefit as much from this course. Then administer Safety Assessment.

This Course will not be suitable for you. *Refer to other sources.* ***Encourage:*** *I really encourage you to go to your GP for a referral to a psychiatrist or clinical psychologist – Medicare does cover 12 sessions per year with these health professionals. OR, other.*

**Safety Assessment: Compulsory for All**

Note, complete relevant sections for all applicants

- If low or no risk (defined as 'yes' to 'a', below), please advise about the following (**and tick**): If mood deteriorates or feels at risk:
  - Contact us
  - Talk to friend
  - BUT if at risk of suicide: Contact emergency/support services if feel suicidal/feel self-harm (Life Line: 13 11 14) or 000.
- If elevated risk (PHQ-9 >9, Qn 9 score >1), complete the following

**“We’ve noted that you had some symptoms of depression OR said that you’ve had thoughts that you would be better off dead.”**

**“A lot of people have difficulty with depression. Some of these people have thoughts of harming themselves”**

| a | In the past 12 months have you had any thoughts that life is not worth living? | No | Yes |
| --- | --- | --- | --- |
| b | In the past 12 months have you had thoughts of harming yourself? | No | Yes |
| c | Do you have any intention or plan of harming yourself? (exclusion criteria) | No | Yes |
| d | In the past 5 years have you ever made any suicide attempts or harmed yourself in anyway? (active suicidal intention (6 months + risk assessment) and recent (12month self harm) = exclusion criteria) | No | Yes |
| If YES to question (c) or (d), exclude, but conduct risk assessment, develop management plan, and document actions and then follow-up with applicant. If NO, continue interview | | | |

- If low or no risk (defined as Yes only to (a)): discuss steps to take if mood deteriorates, as above
- If elevated risk (defined as 'yes' to Qns (c) or (d)), applicant is to be excluded, but conduct risk assessment, develop management plan, and document actions and then follow-up with applicant. If NO, continue interview

**“I’m quite worried about you, it sounds like you are feeling really down. I'd like to ask you more questions**

| **RISK ASSESSMENT** | | | | | | | | |
| --- | --- | --- | --- | --- | --- | --- | --- | --- |
| **Applicant/Participant Name:** | | | | **Date/Time:** | | | | |
| **eCentreClinic Team Member:** | | | | **DOB**: | | **Sex** | |  |
| **Guidelines**   1. **Conduct abbreviated risk assessment** 2. **Develop safety plan in consultation with applicant (document):** 3. **If possible, consult with VirtualClinic clinicians (document)** 4. **Refer to appropriate services: GP, Emergency, Hospital, Lifeline (document)** 5. **Negotiate follow-up with applicant (document)** 6. **Discuss with**  **Principal Investigator (document)** | | | | | | | | |
| **REASON FOR RISK ASSESSMENT (Tick):**  **[ ] Thoughts of harming self in last 12 months**  **[ ] Intention to commit suicide or self-harm?**  **[ ] Stated has made previous attempts at suicide or self harm** | | | | | | | | |
| **SUICIDE** | | | | | | | | |
| **Background factors** | *Y=Yes, N=No, UK=Unknown* | | **Current factors** | | *Y=Yes, N=No, UK=Unknown* | | | |
| Previous suicide attempts | | [ ] Y [ ] N [ ] UK | Recent significant life events | | | | [ ] Y [ ] N [ ] UK | |
| History of other self harm | | [ ] Y [ ] N [ ] UK | Hopelessness/despair | | | | [ ] Y [ ] N [ ] UK | |
| Family history of suicide | | [ ] Y [ ] N [ ] UK | Expressing high levels of distress | | | | [ ] Y [ ] N [ ] UK | |
| Separated/widowed/divorced | | [ ] Y [ ] N [ ] UK | Expressing suicidal ideas | | | | [ ] Y [ ] N [ ] UK | |
| Isolation/lack of role | | [ ] Y [ ] N [ ] UK | Self-harming behaviour | | | | [ ] Y [ ] N [ ] UK | |
| Other *(specify)* | | [ ] Y [ ] N [ ] UK | Current plan/intent | | | | [ ] Y [ ] N [ ] UK | |
|  | |  | Other *(specify)* | | | | [ ] Y [ ] N [ ] UK | |
| **Summary Notes and Safety Plan (by administering Clinician) (Risk, Protective, Previous attempts, Referred, Safety Plan, Information Provided, other)**  ......................................................................................................................................................................................  ......................................................................................................................................................................................  ......................................................................................................................................................................................  ......................................................................................................................................................................................  Name ………………………………. Signature ……………………………….. Date ……………..  Chief Investigator Review/Notes:  ......................................................................................................................................................................................  ......................................................................................................................................................................................  Name ………………………………. Signature ……………………………….. Date …………….. | | | | | | | | |

**IF NOT SUITABLE:**

**Reason:** Because this is a research Course, we have very strict criteria regarding who we can and cannot include in the Course. Unfortunately, you do not meet criteria for the Course at this point in time.

**Encourage:** I really encourage you to go to your GP for a referral to a psychiatrist or clinical psychologist – Medicare does cover 12 sessions per year with these health professionals. OR, other.

IF SUITABLE: Continue interview and administer GAD-7

**Generalized Anxiety Disorder-7 Item Scale (GAD-7)**

**
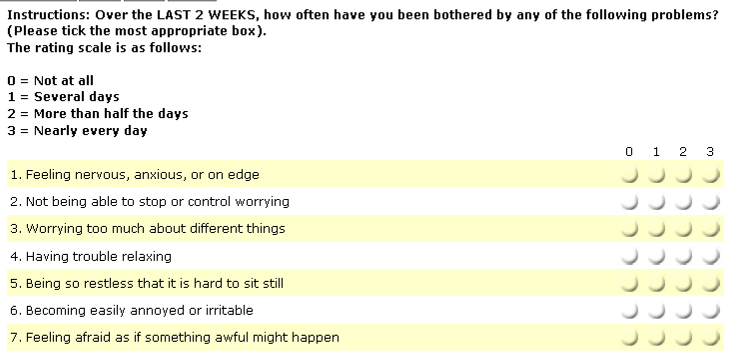
**

**If Total GAD-7 Score <5 then Exit interview, as per above. Else, continue:**

**IF SUITABLE:**

“Thank you for answering those questions. I am really pleased to say that it sounds like the course will be suitable for you, so if would still like to continue I would be very pleased to have you in the course.

The course is a collaboration between the MHA and the eCentreClinic, which is part of the Centre for Emotional Health at Macquarie University. We are testing one of their online courses called the Wellbeing Course. They have tested this with several hundred people and have had good results. We are thinking about using that course as part of our service, so would like people like yourself to help us test it.

We can’t promise that it will cure anyone, but so far 95% of people who have done it say they would recommend it to a friend, so we think it could be really helpful. Let me get some more administrative details out the way.

| a | We need to set up a login user account for you, which I can do. Do you have an email address. We can use that as your login user account. Or, you can set one up and then tell me.  Record ………………………………. | No | Yes |
| --- | --- | --- | --- |
| b | The Wellbeing Course automatically sends emails to tell people about new materials that are available, are you happy for the course to send you that information? Please don’t respond to those emails, they are just there to keep you informed about things. | No | Yes |

**IF ACCEPTED:**

1. **Expectations:** We’ve found that people who get the most out of our Courses spend about 4 hours a week reading the Lessons and completing the homework tasks, and applying the new skills they’re learning on a daily basis.

We also need people to help us by evaluating the courses. We are going to ask you complete questionnaires at various times throughout the Course and it is essential we get everyone’s feedback. We really look forward to hearing from you about how we can improve the Course.

**Does this sound the sort of commitment you can meet?**

1. **Summary:**
   - You can **withdraw** at any time
   - **8 week course**
   - **Starting XXX**
2. We will send you an email to confirm what we have discussed.
3. **Again, congratulations for taking this step towards improving your wellbeing. Thank you for your time.**

**Confirmation Email: To send within 24 hours of successful telephone interview.**

Dear {firstname},

Thank you for your application. We really appreciate the time and effort you took in making your application.

{firstname}, we are pleased to inform you that you have been accepted into the Wellbeing Course. This Course is due to begin on Monday the XX of XX. When the Course begins, you will receive full and immediate access to the online Course materials. And, Linda Manoukian, a Registered Psychologist at the MHA will be in weekly contact with you to support you as you progress through the Course.

{firstname}, in order to keep track of your progress we will ask you to complete questionnaires at different times. We have kept the number of questionnaires to an absolute minimum but it is very important that you complete them. These questionnaires allow us to monitor your progress, to evaluate the Course, and to continue to offer the Course.

On Monday the XX of XX we will send you an email letting you know that the Course is available. After you receive that email, please log in by clicking the “Log In” button on our homepage and complete the questionnaires by Sunday the XX of XX. Completing these questionnaires will give you access to the Course.

{firstname}, congratulations again for taking this important step. We really look forward to having you in the Course.

Kind regards and best wishes,

Ms Linda Manoukian, A/Prof Nickolai Titov, Dr Blake Dear

The Mental Health Association, NSW, and the eCentreClinic, Centre for Emotional Health, Macquarie University

________________________________________________________________________________________________

Please note that email is an insecure communication system. We advise you not to send personal information or information about others. Please note that MHA and eCentreClinic are not a crisis services. In the event of emergency or mental health crisis we advise you to contact the emergency services (Police or Ambulance) on ph 000, or telephone Lifeline on ph 131114, or if available, contact your GP.

This message may contain confidential and/or privileged information.  If you are not the addressee or authorized to receive this for the addressee, you must not use, copy, disclose or take any action based on this message or any information herein.  If you have received this message in error, please advise the sender immediately by reply e-mail and delete this message.  Thank you for your cooperation.
